# Knowledge, attitudes, and practices toward linezolid–serotonergic drug interactions: a cross-sectional study

**DOI:** 10.3389/fphar.2025.1720903

**Published:** 2025-11-25

**Authors:** Atheer Aldairem, Sumaya N. Almohareb, Shuroug A. Alowais, Mohammad S. Shawaqfeh, Abdullah A. Alzahrani, Abdullah Y. Alzahrani, Khalid H. Alqahtani, Khalid Bin Saleh

**Affiliations:** 1 Department of Pharmacy Practice, College of Pharmacy, King Saud Bin Abdulaziz University for Health Sciences (KSAU-HS), Riyadh, Saudi Arabia; 2 King Abdullah International Medical Research Center (KAIMRC), Ministry of National Guard-Health Affairs, Riyadh, Saudi Arabia; 3 Department of Pharmaceutical Care, King Abdulaziz Medical City (KAMC), Ministry of National Guard-Health Affairs, Riyadh, Saudi Arabia; 4 College of Pharmacy, King Saud Bin Abdulaziz University for Health Sciences (KSAU-HS), Riyadh, Saudi Arabia

**Keywords:** linezolid, serotonergic drugs, serotonin syndrome, patient safety, drug–drug interaction

## Abstract

**Background:**

Concomitant use of linezolid with serotonergic agents may lead to serotonin syndrome, a rare but serious complication. This study assessed healthcare providers’ knowledge, attitudes, and practices (KAP) to identify gaps and inform strategies to enhance patient safety.

**Methods:**

We conducted a nationwide, cross-sectional survey among physicians, pharmacists, and nurses practicing in Saudi Arabia. Participants completed a validated questionnaire designed to assess KAP related to linezolid–serotonergic drug interactions. KAP scores were categorized and analyzed using logistic regression to identify predictors associated with higher performance.

**Results:**

Among 116 respondents, 69.0% reported awareness of linezolid–serotonergic drug interaction. However, only 30.2% achieved high knowledge scores (≥6.7/10). Correct identification of serotonin syndrome, Hunter diagnostic criteria, and the recommended 2-week washout period was limited (34.5%, 34.5%, and 41.4%, respectively). Pharmacists had the highest knowledge scores (mean 5.52), followed by physicians (5.18) and nurses (2.79). In multivariable analysis, prior awareness of the interaction (AOR, 23.52; 95% CI, 2.90–190.72) and international training (AOR, 3.51; 95% CI, 1.07–11.53) were associated with higher knowledge scores. Both knowledge and attitude were significantly associated with safer practice behaviors (OR 3.04 and 8.75, respectively; p < 0.05).

**Conclusion:**

Persistent gaps in knowledge and safe prescribing practices related to the linezolid–serotonergic interaction were identified among healthcare providers in Saudi Arabia, reflecting a broader challenge globally. These findings support the need for targeted educational interventions and institution-level policies to improve awareness, enhance patient safety, and reduce preventable harm.

## 1 Introduction

Linezolid is a synthetic oxazolidinone antibiotic that plays a key role in treating serious Gram-positive infections, including methicillin-resistant *Staphylococcus aureus* (MRSA) and vancomycin-resistant *Enterococcus* (VRE) (Hashemian et al., 2018). Beyond its antibacterial properties, linezolid is also a reversible, non-selective monoamine oxidase inhibitor, which can precipitate serotonin syndrome when co-administered with serotonergic medications, such as selective serotonin reuptake inhibitors (SSRIs), serotonin–norepinephrine reuptake inhibitors (SNRIs), tricyclic antidepressants, or certain opioids (Shinabarger, 1999; Taylor et al., 2006).

Serotonin syndrome is a potentially life-threatening condition characterized by the triad of neuromuscular hyperactivity, autonomic instability, and altered mental status (Boyer and Shannon, 2005). Although it is uncommon, its clinical significance is considerable. A recent meta-analysis estimated its overall incidence with linezolid at less than 1%, but the risk nearly doubles when multiple serotonergic agents are used (SanFilippo et al., 2023). Regulatory agencies such as the U.S. Food and Drug Administration (FDA) have issued safety alerts regarding this interaction, yet case reports and pharmacovigilance data continue to document preventable cases of serotonin toxicity associated with linezolid (U.S. Food and Drug Administration, 2011; Lawrence et al., 2006; Bernard et al., 2003).

Awareness of this risk among healthcare providers remains limited. International KAP and survey studies indicate that many clinicians under-recognize serotonin syndrome and lack confidence in its diagnosis and prevention (Prakash et al., 2020; Wali, 2025; Gatti et al., 2021). These knowledge gaps may contribute to unsafe prescribing or delayed recognition, particularly in hospital settings where antimicrobial and psychotropic agents are often co-prescribed.

Despite the clinical significance of this interaction, data from the Middle East, including Saudi Arabia, are limited. To date, there are no published studies assessing the knowledge, attitudes, and practices (KAP) of healthcare providers regarding linezolid–serotonergic drug interactions. This study focuses on a globally underexplored and locally unstudied area, aiming to address knowledge and practice gaps, promote safer prescribing behaviors, and inform institutional stewardship and patient safety.

Accordingly, this study aimed to evaluate the KAP of healthcare providers in Saudi Arabia toward linezolid–serotonergic drug interactions and to identify factors associated with knowledge and practice gaps that could guide future interventions.

## 2 Methods

### 2.1 Study design

This was a cross-sectional study conducted from April 2024 to June 2024 across hospitals nationwide in Saudi Arabia. Survey forms were distributed through virtual platforms. A follow-up reminder was sent to non-respondents after 2 weeks to enhance participation. Because the link was openly accessible and could be forwarded beyond the initial distribution lists, an exact response rate could not be reliably calculated.

The target population included physicians, pharmacists, and nurses working in hospitals across different regions of Saudi Arabia. A non-probability convenience sampling strategy was used to recruit participants using institutional mailing lists, professional WhatsApp groups, and social media platforms targeting healthcare professionals. This approach was selected because the target population, consisting of clinicians with potential exposure to linezolid and serotonergic medications, is relatively specific and not evenly distributed across healthcare settings. Recruitment included physicians, pharmacists, and nurses from multiple institutions to improve diversity and representation. The survey remained open for 3 months, during which 116 complete responses were received. Potential limitations of convenience sampling, including selection bias and unequal participation among professions, were recognized and discussed in the manuscript.

### 2.2 Survey instrument

The survey was developed based on a comprehensive review of published studies, clinical guidelines, and pharmacovigilance resources addressing linezolid–serotonergic drug interactions. Content validity was established through expert review by three consultant clinical pharmacists in infectious diseases and internal medicine who evaluated each item for relevance, clarity and completeness. A pilot test was conducted among ten healthcare professionals who were not included in the final analysis to assess clarity, feasibility, completion time, and face validity. The results of the pilot were further reviewed by three experts, leading to minor modifications in wording and item sequencing.

Internal consistency reliability was confirmed using Cronbach’s alpha (α = 0.80), which reflects acceptable reliability for exploratory KAP surveys.

The survey consisted of four sections designed to evaluate participants’ demographics, knowledge, attitudes, and practices. The first section collected demographic information, including gender, age, region, professional background, years of experience, place of graduation, and whether serotonin syndrome had been encountered before.

The second section assessed knowledge of the linezolid–serotonergic drug–drug interaction (DDI), covering common serotonergic medications, the washout period, diagnostic criteria for serotonin syndrome, symptom recognition, and appropriate management strategies. A response was considered correct if it matched the key answer defined *a priori* based on established clinical guidelines. Knowledge score was calculated using eight items: six single-response questions (scored as one point each) and two multiple-response questions (scored up to two points each), yielding a total possible score of 10.

The third section evaluated attitudes toward the drug interactions between linezolid and serotonergic drugs in terms of importance, management, and available alternative options. This section consisted of eight items rated on a 5-point Likert scale. The final section assessed clinical practice of healthcare providers regarding the drug interaction. Practice levels were classified using five items on a 5-point Likert scale.

For all domains, composite scores were categorized into three interpretive levels (low, moderate, and high) using equal-interval divisions of the total possible score for each section. However, continuous analyses were primary, with categorized results presented secondarily.

Ethical approval for this study was obtained from the Institutional Review Board. Informed consent was obtained electronically from all participants prior to survey completion. Participation was voluntary and anonymous, and no personally identifiable information was collected.

### 2.3 Statistical analysis

Descriptive statistics were used to summarize participant characteristics and study variables. Categorical variables were presented as frequencies and percentages, while continuous variables were expressed as means and standard deviations (SD).

KAP scores were categorized as described above. Associations between KAP levels and sociodemographic characteristics were initially examined using univariate logistic regression. Variables with a p-value <0.25 in univariate models were entered into multivariable logistic regression to identify independent predictors of higher knowledge, attitude, or practice scores. Results were expressed as adjusted odds ratios (AORs) with 95% confidence intervals (CIs). A p-value <0.05 was considered statistically significant.

Associations between knowledge, attitude, and practice domains were further explored using chi-square tests. All analyses were conducted using IBM SPSS Statistics, version 30 (IBM Corp., Armonk, NY, United States) and visuals were generated using GraphPad Prism version 10.6.1 (GraphPad Software, Boston, MA, United States).

## 3 Results

### 3.1 Participant demographics

A total of 116 healthcare professionals participated in this nationwide cross-sectional survey. The majority of participants were female (56.0%). About half of the respondents were aged 30–39 years (46.6%), followed by those under the age of 30 years (25%). Participants represented all regions of Saudi Arabia, with the highest proportion from the Central region (66.4%). Among respondents, physicians comprised the largest professional group (38.8%), followed by pharmacists (31.0%) and nurses (30.2%). Regarding professional experience, the largest subgroup had 6–10 years of experience (31.9%), followed by 1–5 years (25.0%) and more than 15 years (22.4%). Overall, 41.4% of participants studied abroad (Table 1).

**TABLE 1:** Participant demographics and characteristics (n = 116).

Variable	Category	n (%)
Gender	Male	51 (44.0)
	Female	65 (56.0)
Age group	Under 30 years	29 (25)
	30–39 years	54 (46.6)
	40–49 years	25 (21.6)
	50–59 years	5 (4.3)
	60 years or above	3 (2.6)
Saudi Arabia-region	Central	77 (66.4)
	Western	14 (12.1)
	Northern	6 (5.2)
	Eastern	11 (9.5)
	Southern	8 (6.9)
Profession	Physician Consultant Fellow Specialist Resident General Specialty Infectious disease Internal medicine Critical care/Emergency Psychiatry Cardiology Other	45 (38.8) 23 (19.8) 7 (6) 5 (4.3) 8 (6.9) 2 (1.7) 14 (12.6) 9 (7.75) 4 (34.5) 2 (1.7) 2 (1.7) 14 (12.6)
	Pharmacist Clinical pharmacist Hospital pharmacist Community pharmacist Other	36 (31.0) 21 (18.1) 12 (10.3) 2 (1.7) 1 (0.9)
	Nurse	35 (30.2)
Years of experience	<1 year	7 (6)
	1–5 years	29 (25)
	6–10 years	37 (31.9)
	11–15 years	17 (14.7)
	>15 years	26 (22.4)
Studied abroad	Total	48 (41.4)
	Physician	23 (44.4)
	Pharmacist	10 (20.8)
	Nurse	15 (31.2)
Encountered serotonin syndrome before	Yes Secondary to linezolid	32 (27.6) 19 (16.4)
	No	84 (72.4)

Participant demographics and professional characteristics, including gender, age group, region, profession, years of experience, international study background, and prior encounter with serotonin syndrome. Data are presented as frequency (n) and percentage (%).

### 3.2 Knowledge levels and influencing factors

The mean knowledge score was 4.56 ± 2.92 (out of 10), indicating a moderate level of knowledge regarding linezolid–serotonergic drug interactions. As shown in Table 3, only 30.2% of participants achieved high knowledge scores (6.7–10), whereas 37.1% demonstrated moderate knowledge (3.4–6.6) and 32.8% had low knowledge levels (0–3.3).

Participants’ knowledge of linezolid and serotonergic drug interaction according to profession is summarized in Table 2. There was variation in the awareness of this drug interaction across healthcare professionals. Pharmacists demonstrated the highest mean knowledge scores (5.52 ± 2.23), followed by physicians (5.18 ± 2.70), while nurses scored notably lower (2.79 ± 3.11). Overall, 69.0% of participants were aware of the interaction, with awareness highest among pharmacists (91.7%) and physicians (77.8%), compared to only 34.3% of nurses. Similarly, correct identification of serotonin syndrome as the main manifestation was most common among pharmacists (91.7%) and physicians (77.8%) *versus* nurses (34.3%). Knowledge regarding the recommended 2-week washout period before restarting serotonergic agents was limited, with only 41.4% answering correctly. Awareness of diagnostic criteria was also low, as just 34.5% correctly identified Hunter’s diagnostic criteria, and only 50.0% recognized cyproheptadine as the recommended antidote.

**TABLE 2:** Participants’ knowledge of linezolid and serotonergic drug interaction stratified by profession.

Knowledge items	Total (n = 116)	Physicians (n = 45)	Pharmacists (n = 36)	Nurses (n = 35)
Are you aware of the drug interaction between linezolid and serotonergic drugs?				
Yes	80 (69.0)	35 (77.8)	33 (91.7)	12 (34.3)
No	36 (31.0)	10 (22.2)	3 (8.3)	23 (65.7)
What is the manifestation of the linezolid–serotonergic drug interaction?				
Serotonin syndrome*	80 (69.0)	35 (77.8)	33 (91.7)	12 (34.3)
Hypertensive crisis	6 (5.2)	2 (4.4)	1 (2.8)	3 (8.6)
QT prolongation	5 (4.3)	1 (2.2)	1 (2.8)	3 (8.6)
Hepatotoxicity	2 (1.7)	0 (0.0)	0 (0.0)	2 (5.7)
I don’t know	23 (19.8)	7 (15.6)	1 (2.8)	15 (42.9)
What is the recommended duration of the washout period between linezolid and psychiatric serotonergic drugs?				
24 h	9 (7.8)	5 (11.1)	2 (5.6)	2 (5.7)
3 days	6 (5.2)	1 (2.2)	3 (8.3)	2 (5.7)
1 week	8 (6.9)	4 (8.9)	3 (8.3)	1 (2.9)
2 weeks*	48 (41.4)	17 (37.8)	19 (52.8)	12 (34.3)
No need for washout period	3 (2.6)	1 (2.2)	0 (0.0)	2 (5.7)
I don’t know	42 (36.2)	17 (37.8)	9 (25.0)	16 (45.7)
After stopping linezolid, when should serotonergic psychiatric medication be resumed?				
24 h*	29 (25.0)	11 (24.4)	10 (27.8)	8 (22.9)
48 h	17 (14.7)	7 (15.6)	8 (22.2)	2 (5.7)
72 h	14 (12.1)	4 (8.9)	5 (13.9)	5 (14.3)
Start immediately after	9 (7.8)	4 (8.9)	2 (5.6)	3 (8.6)
I don’t know	47 (40.5)	19 (42.2)	11 (30.6)	17 (48.6)
What are the diagnostic criteria used for serotonin syndrome?				
Caplan’s criteria	1 (0.9)	1 (2.2)	0 (0.0)	0 (0.0)
Engel’s criteria	2 (1.7)	1 (2.2)	1 (2.8)	0 (0.0)
Hunter’s criteria*	40 (34.5)	17 (37.8)	10 (27.8)	13 (37.1)
Newman’s criteria	5 (4.3)	1 (2.2)	1 (2.8)	3 (8.6)
I don’t know	68 (58.6)	25 (55.6)	24 (66.7)	19 (54.3)
What is the recommended antidote therapy for serotonin syndrome?				
Cyproheptadine*	58 (50.0)	26 (57.8)	19 (52.8)	13 (37.1)
Acetylcysteine	7 (6.0)	4 (8.9)	1 (2.8)	2 (5.7)
Trihexyphenidyl	7 (6.0)	0 (0.0)	7 (19.4)	0 (0.0)
Protamine sulfate	3 (2.6)	2 (4.4)	0 (0.0)	1 (2.9)
I don’t know	41 (35.3)	13 (28.9)	9 (25.0)	19 (54.3)

Distribution of responses to knowledge-related items stratified by profession. Data are presented as frequency (n) and percentage (%).

Footnote: Correct response based on clinical guidelines. A response was considered correct if it matched the key answer defined *a priori*. Knowledge scores were calculated by assigning 1 point for each correct single-answer item and up to 2 points for multi-response items, with a maximum score of 10. *Asterisks indicate correct responses.

**FIGURE 1:**
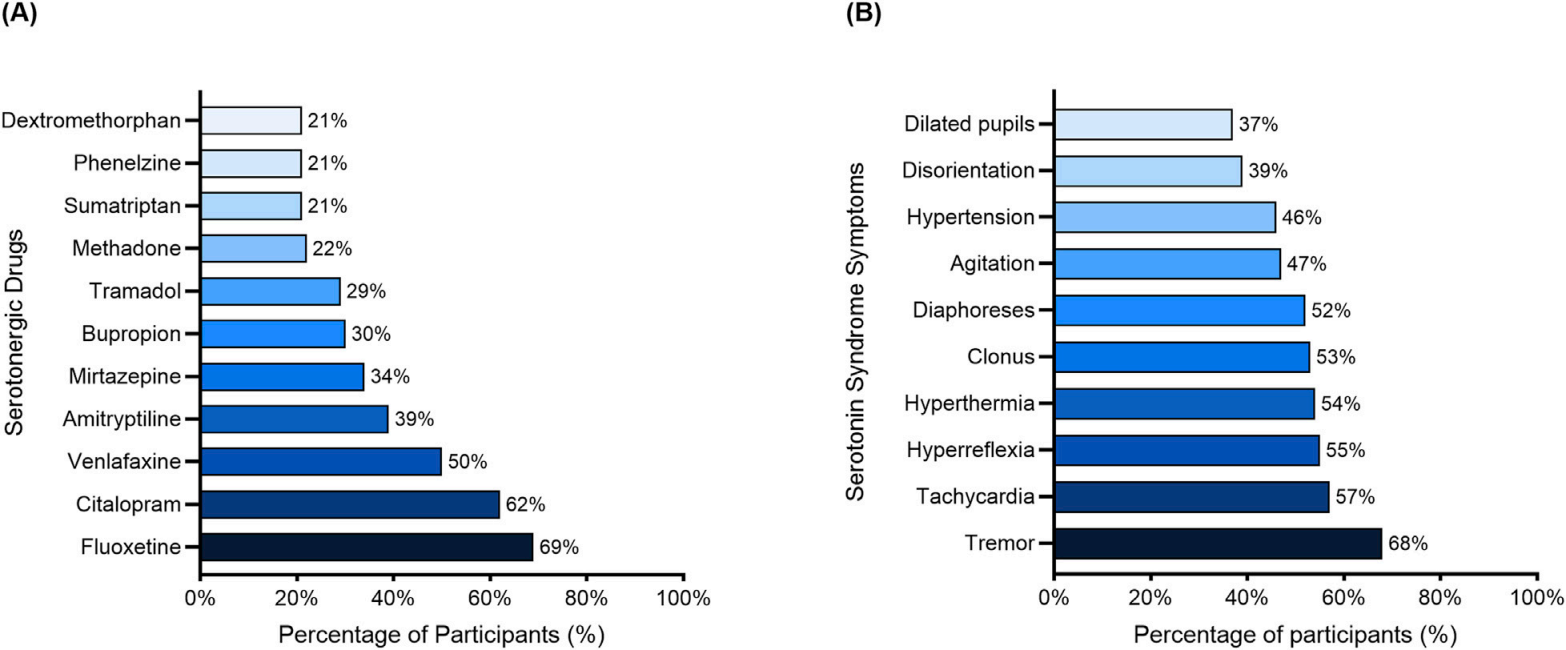
Knowledge of Linezolid–Serotonergic Drug Interaction: Identified Agents and Associated Symptoms. **(A)** Frequency of serotonergic drugs identified by respondents as interacting with linezolid. **(B)** Frequency of identified symptoms associated with serotonin syndrome.

Figure 1A shows that the most frequently recognized serotonergic drugs interacting with linezolid were fluoxetine (68.97%), citalopram (62.07%), and venlafaxine (50%). As shown in Figure 1B, the most frequently identified symptoms of serotonin syndrome were tremor (68%), tachycardia (57%), hyperreflexia (55%), and hyperthermia (54%). However, hallmark signs such as disorientation and dilated pupils were the least frequently identified symptoms.

### 3.3 Attitude and associated factors

The mean attitude score was 3.34 ± 0.43 out of a maximum of 5 (Table 3). Overall, participants acknowledged the clinical importance of the interaction: 53.4% reported feeling confident in identifying drug interactions, 62.1% agreed that the interaction is clinically significant, 55.2% believed that linezolid cannot be safely administered with serotonergic drugs, and another 55.2% agreed that linezolid should only be co-prescribed with serotonergic drugs under limited circumstances and with appropriate monitoring. Meanwhile, 43.1% were neutral whether the serotonergic drug must be immediately discontinued before starting linezolid (Table 4).

**TABLE 3:** Participants’ Knowledge, attitude and Practice Scores and Levels Regarding Linezolid–Serotonergic Drug Interactions.

Domain	Score (mean ± SD)	Score range	Level n (%)		
			High	Moderate	Low
Knowledge	4.56 ± 2.92	0–10	35 (30.2)	43 (37.1)	38 (32.8)
Attitude	3.347 ± 0.347	1–5	8 (6.9)	90 (77.6)	18 (15.5)
Practice	2.84 ± 0.94	1–5	12 (10.3)	62 (53.4)	42 (36.2)

Summary of knowledge, attitude, and practice (KAP) scores and levels among healthcare providers regarding linezolid–serotonergic drug interactions (n = 116).

This table presents the mean (± standard deviation) KAP, scores and their distribution across low, moderate, and high-performance levels. Knowledge was assessed using eight items (score range 0–10), while attitudes and practices were evaluated using items on a 5-point Likert scale.

**TABLE 4:** Participant attitudes toward linezolid–serotonergic drug interaction (n = 116).

Statement	Strongly agree n (%)	Agree n (%)	Neutral n (%)	Disagree n (%)	Strongly disagree n (%)
I Feel confident in my ability to identify potential drug interactions between linezolid and serotonergic drugs	22 (19.0)	40 (34.5)	31 (26.7)	16 (13.8)	7 (6.0)
I Consider the linezolid–serotonergic drug interaction to be clinically significant	34 (29.3)	38 (32.8)	33 (28.4)	9 (7.8)	2 (1.7)
Linezolid can be safely co-administered with serotonergic drugs under any circumstances	3 (2.6)	11 (9.5)	38 (32.8)	39 (33.6)	25 (21.6)
Linezolid can be prescribed to patients taking serotonergic drugs concurrently only if there are limited antibiotic options and the benefit outweigh the risk with close monitoring	13 (11.2)	51 (44.0)	37 (31.9)	11 (9.5)	4 (3.4)
If linezolid must be administered to a patient receiving a serotonergic drug, the serotonergic drug must be immediately stopped before starting linezolid	12 (10.3)	30 (25.9)	50 (43.1)	19 (16.4)	5 (4.3)
In non-emergency situations when non-urgent treatment with linezolid is planned, the serotonergic medication should be stopped at least 2 weeks in advance of linezolid treatment	20 (17.2)	38 (32.8)	48 (41.4)	8 (6.9)	2 (1.7)
I Am aware of the appropriate alternative treatment options when linezolid cannot be used due to the potential interaction with serotonergic drugs	29 (25.0)	42 (36.2)	39 (33.6)	4 (3.4)	2 (1.7)
From the published observational studies, the risk of serotonin syndrome is low when using linezolid concurrently with other serotonergic medication like antidepressants	4 (3.4)	26 (22.4)	61 (52.6)	22 (19.0)	3 (2.6)

Distribution of responses to attitude-related statements among healthcare providers. Data are presented as frequency (n) and percentage (%).

### 3.4 Practice

Practice scores were generally low (mean = 2.84 ± 0.94), with only 10.3% of participants achieving a high level of practice (Table 3). For example, only 34.5% reported always screening for serotonergic medications before prescribing linezolid, and 24.1% always educated patients about the interaction (Table 5). Moreover, 61.2% admitted to previously prescribing or approving linezolid with serotonergic medications without being aware of the interaction, while only 7.8% reported doing so with full awareness.

**TABLE 5:** Practices of healthcare professionals regarding linezolid–serotonergic drug interaction.

Practice statement	Always n (%)	Often n (%)	Sometimes n (%)	Rarely n (%)	Never n (%)
I Routinely screen for serotonergic medications before prescribing/verifying linezolid	40 (34.5)	26 (22.4)	16 (13.8)	15 (12.9)	19 (16.4)
I Routinely educate patients about the interaction between linezolid and serotonergic drugs	28 (24.1)	27 (23.3)	22 (19.0)	14 (12.1)	25 (21.6)
I Collaborate with other healthcare professionals to review medication regimens	45 (38.8)	28 (24.1)	18 (15.5)	9 (7.8)	16 (13.8)
I Previously prescribed/approved linezolid concurrently without awareness of the interaction	4 (3.4)	9 (7.8)	16 (13.8)	16 (13.8)	71 (61.2)
I Previously prescribed/approved linezolid concurrently with full awareness of the interaction	9 (7.8)	11 (9.5)	21 (18.1)	20 (17.2)	55 (47.4)

Distribution of responses to practice-related statements among healthcare providers. Data are presented as frequency (n) and percentage (%).

Supplementary Table 1A summarizes the practice differences between healthcare professionals with and without prior serotonin syndrome encounters. Those with prior exposure were more likely to always screen for serotonergic medications before prescribing linezolid (40.6%). Similarly, 37.5% often collaborated with colleagues to review regimens, and 37.5% routinely educated patients on the interaction. Despite this, gaps remained, with 25% of clinicians with prior encounter admitted to rarely or sometimes prescribing linezolid with serotonergic drugs while unaware of the interaction, and 18.8% reported doing so knowingly.

### 3.5 Association between knowledge, attitude and practice

Chi-square tests and univariate logistic regression showed that knowledge was significantly associated with practice (OR = 3.04; 95% CI, 1.19–7.77; p = 0.021). Similarly, participants with higher attitude scores had significantly greater odds of good practice compared with those with lower attitude scores (OR = 8.75; 95% CI, 2.65–28.89; p < 0.001). However, there was no significant association between knowledge and attitude scores (p = 0.091) (Table 6).

**TABLE 6:** Association between knowledge-practice, knowledge-attitude, and attitude-practice.

Characteristics	Knowledge		Statistics	*p-value*	Univariate logistic regression
	High/Moderate	Low			
Practice					
High/Moderate	28 (24.1%)	46 (39.7%)	χ^2^ = 5.7 df = 1	0.021*	OR = 3.043 95% CI = 1.192–7.773 *p* = 0.02
Low	7 (6%)	35 (30.2%)			
Attitude					
High/Moderate	33 (28.4)	65 (56%)	χ^2^ = 3.674 df = 1	0.091	OR = 4.062 95% CI = 0.881–18.729 *p* = 0.072
Low	2 (1.7%)	16 (13.8%)			
Practice					
High/Moderate	70 (60.3%)	4 (3.4%)	χ^2^ = 15.94 df = 1	<0.001	OR = 8.75 95% CI = 2.65–28.892 *p* < 0.001
Low	28 (24.1%)	14 (12.1%)			

*χ* ^*2*^ *values are from Pearson chi-square tests. Odds ratios (OR) and 95% confidence intervals (CI) were derived from univariate logistic regression*.

Association between knowledge, attitude, and practice levels among healthcare providers. χ^2^ values from Pearson’s chi-square test; odds ratios (OR) and 95% confidence intervals (CI) from univariate logistic regression.

Multivariate regression analyses were conducted to examine the association between sociodemographic characteristics and knowledge, attitude, and practice scores (Supplementary Table 2A). Awareness of the drug between linezolid and serotonergic agents was the strongest predictor across all domains. Participants aware of the interaction had significantly higher odds of achieving better knowledge (AOR = 23.52; 95% CI, 2.90–190.72; p = 0.003), better attitudes (AOR = 20.74; 95% CI, 4.61–93.26; p < 0.001), and better practices (AOR = 8.93; 95% CI, 2.90–27.60; p < 0.001). In addition, participants trained abroad showed significantly higher odds of good knowledge compared with those trained locally (AOR = 3.51; 95% CI, 1.07–11.53; p = 0.039). Years of experience also played a role, where participants with 6–10 years (AOR = 8.47; 95% CI, 1.15–62.42; p = 0.036) and more than 15 years (AOR = 8.77; 95% CI, 1.13–68.14; p = 0.038) demonstrated significantly better practice levels compared to those with less than 1 year of experience. Other demographic factors, including gender, region, and professional background, did not show significant associations in the multivariate analysis.

## 4 Discussion

Serotonin syndrome is a rare but clinically significant and potentially life-threatening adverse drug reaction associated with serotonergic agents (Wali, 2025). Linezolid, a monoamine oxidase inhibitor with broad-spectrum antimicrobial activity, has been implicated in several post-marketing reports and case series as a precipitating agent for serotonin syndrome, particularly when co-administered with serotonergic medications such as selective SSRIs, SNRIs, opioids, and others (Wali, 2025; Gatti et al., 2021; Kufel et al., 2023).

While several retrospective studies suggest that the incidence of serotonin syndrome in real-world settings remains relatively low, this does not diminish its clinical importance. Multiple analysis have found few or no confirmed cases among hospitalized patients receiving linezolid, even in combination with opioid (Kufel et al., 2023; Mitwally et al., 2022; Traver et al., 2022; Yan et al., 2023). In line with these findings, several recent case reports and pharmacovigilance data continue to document serotonin syndrome as a rare yet increasingly observed complication of linezolid use (Corsini Campioli et al., 2020; Masbough et al., 2022; Essakow et al., 2022; Xinpeng et al., 2025). Large-scale safety analyses, such as Novella et al.’s review of 13,312 post-marketing reports, further emphasize the importance of close monitoring in patients receiving linezolid alongside serotonergic medications, especially those with psychiatric comorbidities (Novella et al., 2025).

In our cross-sectional study, healthcare providers demonstrated moderate overall knowledge of the linezolid–serotonergic interaction. Pharmacists had the highest knowledge scores, followed by physicians, while nurses scored the lowest. These differences likely reflect variations in education and clinical roles. Pharmacists receive more in-depth education in pharmacology and drug interaction management, which improves their familiarity with complex drug–drug interactions (Warholak et al., 2011; Abougalambou and Alenezi, 2023). Physicians and nurses, on the other hand, may encounter such interactions less frequently or rely on pharmacists and decision-support alerts for guidance (Lan et al., 2014; Justinia et al., 2021). However, given that participants were recruited using a convenience sampling strategy, some degree of selection bias cannot be excluded. Convenience sampling could have affected the proportional representation of professional groups and resulted in greater participation from individuals with greater access to academic or professional networks (Etikan, 2016). This likely influenced our overall KAP estimates, as they may reflect differences in response opportunity rather than true population-level variation. These factors may limit the generalizability of our findings to broader populations.

Although the majority of respondents were aware of the interaction, a detailed understanding of key clinical elements, such as diagnostic criteria, symptomatology, interacting drug classes, and safe washout periods was limited. Notably, only 34% correctly identified the Hunter Criteria, the most validated diagnostic tool for serotonin syndrome, and less than half recognized hallmark neuromuscular signs such as clonus and hyperreflexia. These findings align with international reports demonstrating variable awareness of serotonin syndrome. In a study among Indian neurologists, only 31% correctly identified the diagnostic criteria for serotonin syndrome, with also low recognition of serotonergic agents and treatment modalities (Prakash et al., 2020). Comparable trends have been reported among Saudi healthcare students and interns, where just over half demonstrated sufficient knowledge about serotonin syndrome (Elashmony et al., 2023). In our study, although common serotonergic agents like fluoxetine and citalopram were frequently recognized, less commonly implicated but equally important agents such as mirtazapine, dextromethorphan, and phenelzine were often missed. These findings across settings highlight a persistent global gap in awareness that warrants targeted education and protocol-based interventions.

Furthermore, only 41% of participants correctly identified the FDA-recommended 2-week washout period before reinitiating serotonergic therapy following linezolid discontinuation (SanFilippo et al., 2023). Similarly, only a quarter of respondents were aware of the proper timing to restart psychiatric serotonergic agents after stopping linezolid, an oversight that can lead to inappropriate and potentially harmful prescribing decisions. Just over 50% of participants recognized cyproheptadine as the preferred antidote for serotonin syndrome, reinforcing the need for greater pharmacotherapy education. These findings are consistent with those reported by Taylor et al. in a retrospective survey, which revealed significant variability in clinician knowledge regarding linezolid–serotonergic drug interactions and the management of serotonin syndrome (Taylor et al., 2006).

Practice patterns reflected a more concerning gap: despite general awareness of the interaction, only 34.5% of respondents reported always screening for serotonergic drugs prior to prescribing linezolid. Alarmingly, 61.2% acknowledged that they had previously prescribed or approved concurrent linezolid and serotonergic medications without awareness of the interaction, and just 7.8% had done so knowingly. This finding has important clinical implications, as inadvertent co-prescription substantially increases the risk of serotonin syndrome, a potentially life-threatening adverse drug reaction that may be misinterpreted as infection-related symptoms. Lack of awareness may also delay recognition and appropriate management, leading to preventable patient harm and unnecessary healthcare utilization. Moreover, such prescribing behavior reflects deficiencies in pharmacovigilance and antimicrobial stewardship practices. These observations highlight the urgent need for targeted educational interventions and the implementation of clinical decision support tools within prescribing systems to enhance clinician awareness and promote safer prescribing practices.

Interestingly, healthcare professionals with prior clinical exposure to serotonin syndrome reported more cautious and collaborative prescribing behaviors (Supplementary Table 1A). For example, 40.6% of exposed clinicians reported always screening for serotonergic medications, compared to 32.1% of those without prior exposure. However, even among those with direct experience, 25% admitted to past prescribing without awareness of the interaction, suggesting that experience alone may not be sufficient to ensure optimal practice, and that systemic educational efforts are warranted.

Contributing to these observed gaps is the lack of clear, standardized guidance regarding the management of this drug-drug interaction. The FDA has issued safety communications highlighting the potential for serious CNS reactions, particularly with SSRIs and SNRIs, but stops short of offering concrete clinical pathways for risk mitigation (U.S. Food and Drug Administration, 2011). Moreover, recent population-based studies indicate that the incidence of serotonin syndrome may be lower than previously assumed, raising questions about the necessity of blanket contraindications (Bai et al., 2022). This ambiguity leaves clinicians navigating a gray zone, often relying on individualized risk-benefit assessments and interdisciplinary consultations, such as consulting psychiatry when considering antidepressant discontinuation or infectious disease (ID) consultation to explore alternative antibiotic options (Morales-Molina et al., 2005).

This study has several limitations. The sample size was relatively limited, reflecting the focused scope of the survey and the challenge of reaching healthcare professionals familiar with linezolid use. The use of convenience sampling and reliance on self-reported practices may introduce recall or reporting bias. In addition, the distribution of responses across professions was uneven, with nurses representing a smaller proportion of respondents compared with physicians and pharmacists, which may have influenced interprofessional comparisons. Nevertheless, the findings provide meaningful insight, and future studies with larger and more systematically sampled populations are warranted.

In conclusion, this study reveals significant gaps between awareness and clinical practices regarding the rare but potentially serious linezolid–serotonergic drug interaction among healthcare professionals in Saudi Arabia. Despite a general awareness of the interaction, inconsistencies in detailed knowledge and safe prescribing behaviors were observed. These findings not only sheds light on a specific issue within the Saudi healthcare context but also raises global awareness of similar challenges that may exist in other regions. To enhance patient safety, this study emphasizes the necessity for targeted educational interventions, the integration of clinical decision support systems, and the reinforcement of institutional protocols. Moving forward, future research should prioritize the development and validation of strategies that effectively bridge the knowledge-to-practice gap, ultimately reducing the risks associated with high-alert antimicrobial prescribing and promoting safer healthcare systems worldwide.

## Data Availability

The original contributions presented in the study are included in the article/Supplementary Material, further inquiries can be directed to the corresponding author.
