# Supplementary material for: Knowledge, attitudes, and practices toward linezolid–serotonergic drug interactions: a cross-sectional study
Source: Front Pharmacol. 2025 Nov 25;16:1720903. doi: 10.3389/fphar.2025.1720903 (PMC12685812; doi:10.3389/fphar.2025.1720903)
Supplement: Supplementary file 1 [file Supplementaryfile1.docx]

**Title: Knowledge, Attitudes, and Practices Toward Linezolid–Serotonergic Drug Interactions: A Cross-Sectional Study**

**Supplementary Appendix:**

**Table 1A. Practice Patterns of Healthcare Professionals with Prior Exposure to Serotonin Syndrome**

|  | **Healthcare professionals with prior exposure to serotonin syndrome** | | |
| --- | --- | --- | --- |
| **Practice Item** | **Total (n = 116)** | **Yes (n = 32)** | **No (n = 84)** |
| **I routinely screen for the serotonergic medications before prescribing or verifying linezolid.** | | | |
| **Never** | 19 (16.4%) | 3 (9.4%) | 16 (19.0%) |
| **Rarely** | 15 (12.9%) | 4 (12.5%) | 11 (13.1%) |
| **Sometimes** | 16 (13.8%) | 3 (9.4%) | 13 (15.5%) |
| **Often** | 26 (22.4%) | 9 (28.1%) | 17 (20.2%) |
| **Always** | 40 (34.5%) | 13 (40.6%) | 27 (32.1%) |
| **I routinely educate patients about the interaction between linezolid and serotonergic drugs.** | | | |
| Never | 25 (21.6%) | 5 (15.6%) | 20 (23.8%) |
| Rarely | 14 (12.1%) | 3 (9.4%) | 11 (13.1%) |
| Sometimes | 22 (19.0%) | 6 (18.8%) | 16 (19.0%) |
| Often | 27 (23.3%) | 12 (37.5%) | 15 (17.9%) |
| Always | 28 (24.1%) | 6 (18.8%) | 22 (26.2%) |
| **I collaborate with other healthcare professionals to review medication regimens and identify potential interactions before initiating linezolid therapy.** | | | |
| **Never** | 16 (13.8%) | 3 (9.4%) | 13 (15.5%) |
| **Rarely** | 9 (7.8%) | 1 (3.1%) | 8 (9.5%) |
| **Sometimes** | 18 (15.5%) | 3 (9.4%) | 15 (17.9%) |
| **Often** | 28 (24.1%) | 13 (40.6%) | 15 (17.9%) |
| **Always** | 45 (38.8%) | 12 (37.5%) | 33 (39.3%) |
| **I previously prescribed or approved linezolid to be used concurrently with serotonergic medication like SSRI antidepressants while not aware about this interaction.** | | | |
| **Never** | 71 (61.2%) | 16 (50.0%) | 55 (65.5%) |
| **Rarely** | 16 (13.8%) | 8 (25.0%) | 8 (9.5%) |
| **Sometimes** | 16 (13.8%) | 3 (9.4%) | 13 (15.5%) |
| **Often** | 9 (7.8%) | 4 (12.5%) | 5 (6.0%) |
| **Always** | 4 (3.4%) | 1 (3.1%) | 3 (3.6%) |
| **I previously prescribed or approved linezolid with full awareness about this interaction to be used concurrently with serotonergic medication like SSRI antidepressants.** | | | |
| **Never** | 55 (47.4%) | 12 (37.5%) | 43 (51.2%) |
| **Rarely** | 20 (17.2%) | 8 (25.0%) | 12 (14.3%) |
| **Sometimes** | 21 (18.1%) | 5 (15.6%) | 16 (19.0%) |
| **Often** | 11 (9.5%) | 6 (18.8%) | 5 (6.0%) |
| **Always** | 9 (7.8%) | 1 (3.1%) | 8 (9.5%) |

**Table 2A. Association between sociodemographic characteristics with knowledge, attitude and practice**

| **Demographic characteristic** | **Knowledge score** | | | | | | **Attitude score** | | | | | | **Practice score** | | | | | |
| --- | --- | --- | --- | --- | --- | --- | --- | --- | --- | --- | --- | --- | --- | --- | --- | --- | --- | --- |
|  | **Univariate logistic** | | | **Multivariate logistic** | | | **Univariate logistic** | | | **Multivariate logistic** | | | **Univariate logistic** | | | **Multivariate logistic** | | |
|  | **COR** | **95% CI** | **p-value** | **AOR** | **95% CI** | **p-value** | **COR** | **95% CI** | **p-value** | **AOR** | **95% CI** | **p-value** | **COR** | **95% CI** | **p-value** | **AOR** | **95% CI** | **p-value** |
| **Gender** |  |  |  |  |  |  |  |  |  |  |  |  |  |  |  |  |  |  |
| Male | 1.0 |  |  |  |  |  | 1.0 |  |  |  |  |  | 1.0 |  |  |  |  |  |
| Female | 0.904 | 0.4-2 | 0.803 |  |  |  | 0.78 | 0.28-2.18 | 0.637 | - | - | - | 0.425 | 0.191-0.943 | 0.035* | 0.985 | 0.317-3.06 | 0.979 |
| **Age** |  |  |  |  |  |  |  |  |  |  |  |  |  |  |  |  |  |  |
| Under 30 | 1.0 |  |  |  |  |  | 1.0 |  |  |  |  |  | 1.0 |  |  |  |  |  |
| 30–39 | 3.393 | 1.02-11.2 | 0.045* | 5.569 | 0.476-65.177 | 0.171 | 3.12 | 0.227-43.02 | 0.395 | - | - | - | 1.93 | 0.757-4.922 | 0.169 | - | - | - |
| 40–49 | 4.91 | 1.31-18.35 | 0.018* | 7.579 | 0.467-1.22 | 0.154 | 2.5 | 0.2-30.6 | 0.473 | - | - | - | 1.44 | 0.48-4.32 | 0.511 | - | - | - |
| 50–59 | 0.11 | 0.008-1.473 | 0.096 | 0.148 | 0.006-3.577 | .240 | 3.67 | 0.25-53.83 | 0.343 | - | - | - | 1.219 | 0.17-8.42 | 0.841 | - | - | - |
| 60 or above | 3.12 | 0.227-43.02 | 0.394 |  |  |  | 2 | 0.08-51.6 | 0.676 | - | - | - | 0.406 | 0.03-4.99 | 0.482 | - | - | - |
| **Profession** |  |  |  |  |  |  |  |  |  |  |  |  |  |  |  |  |  |  |
| Physician | 1.0 |  |  |  |  |  | 1.0 |  |  |  |  |  | 1.0 |  |  |  |  |  |
| Pharmacist | 1.25 | 0.495-3.16 | 0.635 |  |  |  | 1.88 | 0.621-5.68 | 0.264 | 1.895 | 0.298-12.047 | 0.498 | 1.35 | 0.507-3.623 | 0.545 | 1.93 | 0.548-6.83 | 0.305 |
| Nurse | 0.656 | 0.239-1.8 | 0.414 |  |  |  | 5.89 | 1.17-29.59 | 0.031* | 1.821 | 0.466-7.121 | 0.389 | 0.38 | 0.152-0.951 | 0.039* | 1.176 | 0.306-4.515 | 0.814 |
| **Region** |  |  |  |  |  |  |  |  |  |  |  |  |  |  |  |  |  |  |
| Central | 1.0 |  |  |  |  |  | 1.0 |  |  |  |  |  | 1.0 |  |  |  |  |  |
| Western | 0.883 | 0.252-3.1 | 0.846 | - | - | - | 0.417 | 0.11-1.56 | 0.195 |  |  |  | 0.762 | 0.24-2.42 | 0.762 | .617 | .124-3.085 | .557 |
| Northern | 0.442 | 0.049-3.98 | 0.467 | - | - | - | 0.83 | 0.09-7.82 | 0.873 |  |  |  | 2.85 | 0.32-25.7 | 0.349 | 5.906 | .449-77.651 | .177 |
| Eastern | 1.84 | 0.511-6.62 | 0.351 | - | - | - | 0.75 | 0.14-3.94 | 0.734 |  |  |  | 0.476 | 0.13-1.7 | 0.254 | .625 | .124-3.141 | .569 |
| Southern | 0.315 | 0.037-2.7 | 0.293 | - | - | - | a |  |  |  |  |  | 4 | 0.468-34-206 | 0.205 | 9.981 | .491-202.94 | .134 |
| **Years of experience** |  |  |  |  |  |  |  |  |  |  |  |  |  |  |  |  |  |  |
| <1 year | 1.0 |  |  |  |  |  | 1.0 |  |  |  |  |  | 1.0 |  |  |  |  |  |
| 1–5 years | 0.278 | 0.047-1.648 | 0.158 | 0.092 | 0.007-1.210 | 0.070 | 1.19 | .107-13.3 | .887 | - | - | - | 1.89 | 0.35-10.02 | 0.455 | 5.3 | 0.76-36.76 | 0.091 |
| 6–10 years | 0.705 | 0.143-3.47 | 0.668 | - | - | - | .738 | .076-7.147 | .793 | - | - | - | 3.15 | 0.6-16.49 | 0.174 | 8.470 | 1.15-62.42 | .036* |
| 11–15 years | 0.629 | 0.336-10.463 | 0.474 | - | - | - | .667 | .058 -7.635 | .744 | - | - | - | 1.905 | 0.32-11.31 | 0.478 | 3.215 | .412-25.097 | .265 |
| >15 years | 0.741 | 0.141-3.88 | 0.722 | - | - | - | .600 | 0.06-6.05 | 0.665 | - | - | - | 3 | 0.54-16.64 | 0.209 | 8.77 | 1.13-68.14 | 0.038* |
| **Training location** |  |  |  |  |  |  |  |  |  |  |  |  |  |  |  |  |  |  |
| Local | 1.0 |  |  |  |  |  | 1.0 |  |  |  |  |  | 1.0 |  |  |  |  |  |
| Abroad | 3.58 | 1.56-8.204 | 0.0.003 | 3.51 | 1.068-11.533 | 0.039* | 0.86 | 0.313-2.37 | 0.774 | - | - | - | 0.909 | 0.42-1.96 | 0.808 | - | - | - |
| **Aware of DDI** |  |  |  |  |  |  |  |  |  |  |  |  |  |  |  |  |  |  |
| No | 1.0 |  |  |  |  |  | 1.0 |  |  |  |  |  | 1.0 |  |  |  |  |  |
| Yes | 25.87 | 3.375-198.276 | <0.002 | 23.515 | 2.895-190.72 | 0.003* | 18.3 | 0.48-69.33 | <0.001* | 20.74 | 4.614-93.258 | <0.001* | 8.422 | 3.46-20.48 | <0.001* | 8.928 | 2.9-27.6 | <.001* |
| **Encountered SS in practice** |  |  |  |  |  |  |  |  |  |  |  |  |  |  |  |  |  |  |
| No | 1.0 |  |  |  |  |  | 1.0 |  |  |  |  |  | 1.0 |  |  |  |  |  |
| Yes | 2.33 | 0.992-5.489 | 0.052 | 1.351 | 0.468-3.895 | 0.578 | 0.98 | 0.32-3.04 | 0.984 | - | - | - | 2.55 | 0.99-6.55 | 0.053 | 2.287 | 0.7-7.44 | 0.17 |

**Footnote:** *a: excluded from interpretation due to sparse data. ss: serotonin syndrome*
